# Understanding how the Support Needs Approach for Patients (SNAP) enables identification, expression and discussion of patient support needs: A qualitative study

**DOI:** 10.1177/17423953211047840

**Published:** 2021-10-22

**Authors:** A Carole Gardener, Gail Ewing, Christi Deaton, Morag Farquhar

**Affiliations:** 1 School of Health Sciences, 6106 University of East Anglia, Norwich, UK; 2 Centre for Family Research, 2152 University of Cambridge, Cambridge, UK; 3 Department of Public Health and Primary Care, 2152 University of Cambridge School of Clinical Medicine, Cambridge, UK

**Keywords:** Person-centred care, long-term conditions, support need, clinical intervention

## Abstract

**Objectives:**

To identify whether and how the support needs approach for patients enables patients with chronic progressive conditions to identify, express and discuss their unmet support needs.

**Methods:**

Thirteen healthcare professionals trained in the Support Needs Approach for Patients (SNAP), recruited from three pilot sites in the East of England (across primary, community and secondary care) delivered SNAP to 56 patients with the exemplar condition chronic obstructive pulmonary disease over a 4-month period. Healthcare professionals participated in a mid-pilot semi-structured interview (pilot site representatives) and end-of pilot focus group (all healthcare professionals). Twenty patients who received SNAP were interviewed about their experiences (topic-guided). Transcripts analysed using a framework approach.

**Results:**

There were differences in how healthcare professionals delivered SNAP and how patients engaged with it; analysing the interaction of these identified a continuum of care (from person-centred to healthcare professional-led) which impacted patient identification and expression of need and resulting responses. When delivered as intended, SNAP operationalised person-centred care enabling patient-led identification, expression and discussion of support needs.

**Discussion:**

SNAP addresses the rhetoric within policy, good practice guidance and the person-centred care literature espousing the need to involve patients in identifying their needs and preferences by providing healthcare professionals with a mechanism for achieving holistic person-centred care in everyday practice.

## Introduction

The need to involve patients with progressive and chronic conditions in identifying and discussing where they need more support to manage life with their conditions (their support needs) is widely agreed.^1,2^ Patient involvement is key to delivering holistic, person-centred, supportive care in which decisions are led by patients’ values, preferences and needs.^1–3^

Recommended approaches to involving patients in identifying and discussing their support needs usually comprise assessment followed by personalised care planning, typically supported by tools completed with, or by, the patient.^4,5^ However, such approaches do not necessarily enable patients to identify and express where they need more support, nor to do so comprehensively. Most tools underpinning these approaches focus on the identification of symptoms, illness burden and problems^6–8^; although these can be useful *indicators* of need, they do not *directly* enable patients to comprehensively identify and communicate their priorities regarding support they need.^9,10^ Furthermore, healthcare professionals (HCPs) sometimes use these *indicators* of need within their assessments of individuals’ needs rather than using an approach enabling patients to prioritise and discuss issues that currently matter to them (i.e. *direct* consideration of their unmet support needs).^11^

The Support Needs Approach for Patients (SNAP)^12,13^ provides an alternative to such HCP-led indirect approaches. Modelled on the internationally recognised evidence-based Carer Support Needs Assessment Tool Intervention (CSNAT-I) for informal carers,^14,15^ SNAP is an intervention which operationalises delivery of holistic person-centred care for patients with chronic or progressive conditions. SNAP comprises a concise evidence-based validated tool (a set of 15 questions: the ‘SNAP Tool’) to help patients *directly* consider areas where they need (more) support, which then informs a needs-led conversation between patient and HCP to identify, express, prioritise and address their unmet support needs.

A pilot study conducted with an exemplar population (people with chronic obstructive pulmonary disease; COPD) explored the delivery and utility of SNAP in clinical practice, capturing views of patients who experienced the intervention and HCPs delivering it. This paper reports the findings relating to whether, and how, SNAP supported patients to identify and express their unmet support needs. A separate paper will focus on HCPs’ experiences implementing SNAP.

## Methods

A qualitative approach was taken, addressing two research questions:

1. How do patients and HCPs engage with SNAP to identify and manage their unmet support needs?
2. What factors enable, or hinder, patient-led identification and expression of support needs through delivery of SNAP?

The study design was peer-reviewed via the study funder (Marie Curie) and reviewed and approved by the North West-Preston Research Ethics Committee (REC reference 18/NW/0234).

### The SNAP intervention

Box 1 summarises the key elements of SNAP using items 1–9 of the Template for Intervention Description and Replication (TIDieR) checklist and guide^16^; items 10–12 (i.e. how the intervention was delivered within an empirical study) are not included in the box but reported in the text below.

Box 1. Key elements of SNAP summarised according to TIDieR^16^ (items 1–9).

**Table:** 

TIDieR guideline item	The Support Needs Approach for Patients (SNAP)
**1. Brief description**	The Support Needs Approach for Patients (SNAP) is an intervention which seeks to enable person-centred care for patients with chronic or progressive conditions. SNAP uses an evidence-based validated tool (the SNAP Tool) to help patients identify and express their support needs which are then discussed with their practitioner in a needs-led conversation.
**2. Why:** Rationale, theory and goal of the elements essential to the intervention	Patients have unmet support needs which, for a variety of reasons, they do not always articulate.^22–26^ A person-centred approach could help with this given that the principle of person-centred care is that patients are perceived as partners in their own health and health care, and that the person should be the focus, not their illnesses or conditions.^3^ The Picker Institute has argued that key to achieving this approach is via ‘understanding and respecting peoples values, preferences and expressed needs’,^27^ however there is little guidance on how to achieve this in practice, thereby compromising the person-centred focus of many healthcare models. SNAP was informed by, and developed to operationalise, the principle of person-centred care through a five-stage approach designed to support patient-led identification, expression and discussion of where they feel they need more support (their expressed need). The five stages of SNAP, and rationale for each, are outlined below:
	Stage 1 – Introduction of the SNAP Tool: patients are introduced to the SNAP Tool by the practitioner, in which (to encourage patient engagement) it is referred to as the ‘How Are You? booklet’. The goal here is to facilitate patient perceptions of legitimacy in relation to expressing their support needs to the practitioner and the comprehensive focus of the intervention.
	Stage 2 – Consideration of needs: using the SNAP Tool, the patient considers their needs and then completes the tool. Both the design of the SNAP Tool and the emphasis on patient-completion of the tool seek to enable patient visualisation of a comprehensive set of broad areas of support need (domains; presented on the SNAP Tool as questions) and enable patient consideration of their particular support needs within those domains. Self-completion of the tool also enables patients to indicate to the practitioner the domains where they need more support, and to prioritise the support need domain most important to them at that point in time.
	Stage 3 – Needs-led conversation between the patient and practitioner: this is a conversation between the patient and practitioner to enable patients to express, and further explore, their individual support needs within their prioritised support domain.
	Stage 4 – Shared response: the patient and practitioner together tailor responses to the patient-prioritised need(s). The goal is to provide an opportunity for the patient and practitioner to identify what supportive input would be valuable and is accessible, and create a shared action plan, drawing on the resources available to both the patient and the practitioner.
	Stage 5 – Shared review: the patient and practitioner, together, review the outcome of the shared response(s) and consider when the five-stage approach might commence again. The rationale for recommencing the five stages again is that patients’ support needs change. There may be times when a full revisiting of the patient's support needs is beneficial (e.g. deterioration in their condition, change in their care plan or informal support arrangements); the prompt for a review may therefore come from the patient or practitioner.
**3. What – intervention materials**	Essential materials for SNAP:
	SNAP Tool: this is a designed-for-purpose, validated tool comprising 15 evidence-based questions (each relating to a broad domain of support need) to help patients consider areas where they may need more support. This is an essential component of the intervention but is only a component – as outlined by the five stages above, there is more to SNAP than the SNAP Tool. The tool is provided once a licence is in place. The tool can be translated, with permission (under a specific translation licence), into different languages.
	SNAP Tool licence: the SNAP Tool is protected by copyright, and a licence required for its use (free for not-for-profit organisations). This can be requested via the SNAP website at https://thesnap.org.uk/use-snap/licensing/ once SNAP training has been completed.
	SNAP training: all practitioners who deliver SNAP must complete SNAP training. Training is currently available in two formats: (1) online via the SNAP website at https://thesnap.org.uk/use-snap/training/ (narrated PowerPoint with downloadable workbook; 90 min to complete; no cost) or (2) bespoke face-to-face training which can be delivered as a train the trainer model (typically a 1-day workshop bringing together a small group of practitioners plus three post-workshop discussion sessions to share experiences and problem solve; price depends on the agreed training package).
	Optional materials for SNAP:
	SNAP support plan: this optional document/framework can be used for recording SNAP activity and outcomes in clinical records as the SNAP Tool itself cannot be reproduced in clinical records (as per the licence). Furthermore, the support plan is more meaningful in terms of reporting SNAP actions and outcomes than the tool itself (the tool is just the conversation starter). The SNAP support plan is provided with the SNAP Tool once a licence is in place.
	SNAP website: the SNAP website is the portal for accessing information about SNAP including SNAP training, licencing and resources (including SNAP publications): https://thesnap.org.uk/
**4. What – intervention procedures**	SNAP comprises five stages summarised below but outlined in more detail with their rationale in item 2: Stage 1 – Introduction of the SNAP Tool: patients are introduced to the SNAP Tool by the practitioner. Stage 2 – Consideration of needs: using the SNAP Tool, the patient considers their needs and completes the tool. Stage 3 – Needs-led conversation between the patient and practitioner: a conversation between the patient and practitioner to prioritise their support needs using their self-completed tool. Stage 4 – Shared response: the patient and practitioner together tailor responses to the patient-prioritised need. Stage 5 – Shared review: the patient and practitioner, together, review the outcome of the shared response(s) and consider when the five-stage approach might recommence.
**5. Who provided**	SNAP is practitioner neutral: it does not prescribe who the intervention should be provided by, however all SNAP providers must have completed SNAP training (accessible at: https://thesnap.org.uk/use-snap/training/) and a SNAP Tool licence for clinical practice use must be in place.
**6. How**	SNAP is condition neutral: although originally designed for patients with COPD, empirical evidence^12^ has informed its application across a range of chronic or progressive conditions. The five stages were designed for delivery to individual patients and it is essential that the practitioner delivering the intervention is SNAP-trained. The mode of delivery is not prescribed. SNAP was designed with face-to-face delivery in mind, however a key feature of SNAP's design is the deliberately limited prescription on how the five-stages are delivered (beyond core elements specified in the training); this is in order to enable fit with(in) existing practice(s). Anecdotal reports of more recent implementation demonstrate remote delivery of the intervention, either in its entirety or for just some stages.
**7. Where**	SNAP is setting neutral: designed to enable delivery in a range of settings. To date, it has been used in primary care, community care, secondary care and hospice day care. There is potential for delivery across settings where practitioners in both settings are SNAP-trained e.g. Stage 1 could be delivered in secondary care prior to discharge, with stages 3–5 facilitated by community practitioners.
**8. When and how much**	When and how often SNAP is delivered is not prescribed, however it should not be considered a one-off intervention and delivery of all five stages are required. Most commonly the five stages would be delivered over three or more patient-practitioner contacts (e.g. Stage 1 at first contact, Stages 3 and 4 at second contact, then Stage 5 at third contact), however delivery over two contacts is possible (e.g. Stage 1 conducted postally followed by the remaining stages as above, or stages 1–4 at first contact, then Stage 5 at second contact). SNAP delivery could occur at any point in a patient's trajectory, depending on their need, the nature of their condition and the clinical setting. For example, in primary care delivery typically occurs in conjunction with patients’ annual reviews for their chronic condition. Delivery of SNAP is also a cycle – it is repeated because patients’ support needs change. As noted above, the prompt to repeat the five stages may come from the patient or practitioner, may be reactively triggered by an event, or proactively planned for.
**9. Tailoring:** If the intervention was planned to be personalised, titrated or adapted, then describe what, why, when, and how	As an intervention to enable person-centred care, and one designed to fit within existing services, SNAP is flexible – for the patient, the practitioner and the service context. Hence, there are core delivery elements (specified in the training), but other elements delivery can be tailored e.g. the practitioner (see item 5), the patient population (see item 6), the mode of delivery (see item 6) and the clinical setting (see item 7).

COPD, chronic obstructive pulmonary disease.

### Pilot sites: recruitment and training

East of England pilot sites were sought in primary, community and secondary care via research engagement events. Four teams were recruited: one primary care practice, two community specialist respiratory teams and one secondary care respiratory team. One community specialist team did not go on to deliver SNAP in practice and therefore is not included in this analysis; their experience of attempting to implement SNAP is reported in the separate implementation paper.

In the remaining sites, HCPs and support staff linked to the care of patients with COPD were sent study recruitment packs (directly or via team lead) including an invitation letter, participant information sheet and details for replying to the research team (reply slip and pre-paid envelope, email and telephone number). Responding HCPs were contacted by the researcher (CG), given the opportunity to ask further questions and arrangements made for their pre-pilot workshop.

Five setting-specific pre-pilot workshops were conducted within teams’ administrative bases and involved 20 participants: 15 HCPs, two nursing students and three support/administrative staff. Workshops were dual purpose: (1) delivering SNAP training and (2) data-generating (regarding their understanding of person-centred care and existing approaches to identifying and responding to patient support needs). Participating staff completed a consent form and brief background information questionnaire. Each workshop was co-facilitated by two of the authors (MF and CG), lasted approximately 2 h and audio-recorded (with permission). The training component included the principles of person-centred care, why and how SNAP was developed, SNAP's five stages and how SNAP could be implemented in their clinical setting. Thirteen of the 15 SNAP-trained HCPs then delivered SNAP in the pilot sites (the two medics did not).

### Four-month pilot implementation

The 4-month pilot period varied by setting, between July 2018 and January 2019. To facilitate delivery, patient inclusion/exclusion criteria were suitably pragmatic: the target population was adults diagnosed with COPD and only those unable to provide informed consent were excluded. As SNAP is designed with flexibility to fit with existing practice, SNAP-trained HCPs could determine three delivery factors for their respective settings: (1) the service delivery context(s), (2) targeted patient sub-groups (within the pilot's inclusion criterion) and (3) patient approach method (SNAP Stage 1). Box 2 shows the outcomes of these determinations.

Box 2. Site-determined pilot delivery factors for their specific settings

**Table:** 

Participating site	Delivery context	Target patient group	Patient approach (SNAP stage 1)
Primary care	Delivered within usual care i.e. COPD annual reviews	Patients due for COPD annual review delivered by the practice's specialist respiratory nurse	Recruitment packs (including the SNAP Tool) were sent out with usual postal invitation to attend for COPD annual review
Community: specialist respiratory team 1	Delivered within usual care i.e. home visits	Patients with COPD due to receive a home visit from a member of the community specialist respiratory team	Recruitment packs (including the SNAP Tool) were given to patients by HCP during a home visit
Secondary care: respiratory department	Delivered in pilot-specific consultation with specialist respiratory nurse or within usual care (i.e. secondary care outpatient appointment)	Patients with COPD due to attend an outpatients’ appointment with specialist respiratory nurses	Patients were first contacted by phone by a research nurse to ascertain whether the patient was willing to consider taking part – those agreeing were then sent a recruitment pack (including the SNAP Tool) by post
	Delivered within usual care	In-patients with COPD identified for discharge via the Early Supported Discharge Service (ESDS)	Patients were given a recruitment pack (including the SNAP Tool) by an ESDS HCP prior to discharge

COPD, chronic obstructive pulmonary disease; HCP, healthcare professional; SNAP, Support Needs Approach for Patients.

The pilot sample size was similarly pragmatic: as the first delivery of SNAP in clinical practice the learning could then inform any required future formal SNAP sample size calculations. To enable HCPs to invite eligible target patients to participate, each site was provided with patient recruitment packs (invitation letter from the clinical team, printed on their headed paper; participant information sheet; consent form and a SNAP Tool, for patient self-completion as part of the intervention). The invitation letter represented Stage 1 of SNAP, explaining the tool's purpose and how it would be used in the follow-on appointment, however how the pack was distributed varied by service (see Box 2). Patients then self-completed their tool (Stage 2) and the remaining stages of the intervention were then delivered by HCPs to 56/58 consenting patients (Stages 3–5): non-delivery of intervention to two patients (who had consented) was due to patient death and time constraints. Box 3 presents patient response rates.

### HCP interviews and pilot-end focus groups

HCPs representing each site (*n* = 1–2/site) participated in mid-pilot topic-guided individual/group interviews either in-person or by telephone (with CG). The topic guide (informed by Normalisation Process Theory^17^) covered implementation processes (reported separately) and SNAP use in clinical practice (reported here). The interviews also monitored and addressed pilot factors: SNAP Tool use, patient recruitment and SNAP delivery.

Pilot-end focus groups involving 11 of the 13 HCPs (plus the two medics who did not deliver SNAP), were co-facilitated by MF and CG, lasted approximately an hour, and took place within each participating site's administrative base. Two additional interviews were conducted with a community team lead and primary care practice manager due to their unavailability for the focus groups. The topic guide, again informed by Normalisation Process Theory, covered HCP experiences of SNAP training, preparing for delivering SNAP within their clinical setting and using SNAP in their clinical practice. Only data on using SNAP in their clinical practice contributed to analyses reported here.

### Patient interviews

All 56 patients who received the intervention during the pilot study were invited to be interviewed about their experience of SNAP. With patients’ written consent, each clinical setting provided the research team with eligible patients’ names and addresses, copies of their consent forms and patient-completed SNAP Tools. The research team then sent each patient a second recruitment pack inviting them for interview (pack comprised of a letter of invitation, participant information sheet and details for replying to the research team: reply slip and pre-paid envelope, email and telephone number). Those who responded were then contacted by telephone to answer any questions and, if happy to proceed, arrangements were made for the interview to take place in their chosen location. Twenty patients agreed to take part (see Box 3) comprising 10 men and 10 women whose ages ranged 53–87 years; this is within the typical demographic profile of people with COPD^18^; however, as a qualitative study our goal is transferability rather than representativeness.^19^ HCPs reported that patients were from across the range of COPD stages and had a number of co-morbidities.

Box 3. Patient recruitment to SNAP by site

**Table:** 

Activity	Primary	Community	Secondary	Total	Target
Pilot start	Mid-July 2018	1st September 2018	1st October 2018	n/a	n/a
Pilot end	Mid-December 2018*	31st December 2018	31st January 2019	n/a	n/a
No. of packs distributed to sites	67	100	60	227	n/a
No. of packs given to patients	67	64	41	172	n/a
No. of patients consenting to receive SNAP	13	20	25	58	n/a
No. of patients who received SNAP	13	20	23	56	n/a
No. of mid-pilot HCP interviews	3	3	2	8	n/a
No. of patients interviewed	9	5	6	20	18–24

*Five months due to 1-month annual leave by HCP.

HCP, healthcare professional; SNAP, Support Needs Approach for Patients.

Semi-structured qualitative interviews were conducted by CG (usually in patients’ homes). The topic guide covered living with COPD, their usual care, impressions of the SNAP Tool (e.g. ease of use and relevance to people with COPD), their rationale for identifying their areas of support need on the SNAP Tool and experience of participating in SNAP (the intervention).

### Data processing and analysis

All patient and HCP interviews/focus groups were audio-recorded (with permission), transcribed by a professional transcription service, checked for accuracy and anonymised. Transcripts were analysed using a framework approach,^20^ facilitated by NVIVO.^21^ An initial framework was developed by CG from key issues arising from both the data and the topic guide. A subsequent coding framework was agreed by all co-authors to guide further analysis, organise the data and develop the final themes.

### Patient and public involvement

The pilot sat within an on-going research programme supported by a patient and public involvement (PPI) advisory group. For the pilot study, PPI members: (1) reviewed participant recruitment documents’ appropriateness and clarity, (2) contributed to SNAP training development and (3) reviewed (endorsing) the thematic analysis findings.

## Results

Results are reported in two sections. The section ‘Approaches to and engagement with SNAP’ describes HCP approaches to SNAP and patient engagement with it; the section ‘Types of care’ then explores the interaction between HCP approaches and patient engagement to characterise types of care enacted and their impact.

### Approaches to and engagement with SNAP

#### Theme 1. HCP approaches to SNAP delivery

HCPs either delivered SNAP as planned (demonstrating intervention fidelity) or deviated from the intended delivery (demonstrating limited intervention fidelity) with subsequent consequences.

##### Delivering SNAP as planned

From the outset approximately half the HCPs recognised SNAP's potential to support delivery of person-centred care. One HCP recounted that she: ‘could see the value it [SNAP] would add and the difference it would make to our appointments…because we were looking at what was important to the patient particularly around whether or not there were non-medical needs’ (HCP011). These HCPs went on to deliver SNAP as planned (demonstrating fidelity). This was clear in both patient and HCP accounts which illustrated how: (1) patients were enabled to complete the SNAP Tool themselves, (2) their tool responses then informed the SNAP conversation and (3) HCPs enabled patient involvement in developing the resulting shared response to their identified needs.

Their narratives also demonstrated additional strategies these HCPs used to further enable the person-centred focus of SNAP. Firstly, there were examples of HCPs personally introducing SNAP to patients and using this to highlight the opportunity SNAP provided for patients to discuss ‘some of the things that you might be worried about that we may not have asked you about’ (HCP011) or ‘look at what your priorities are’ (HCP013). Secondly, patients frequently described HCP actions that actively supported patient participation in SNAP Stages 3 and 4 by asking open questions, displaying empathy, offering suggestions and giving consideration to patients’ views. For example, one patient described their HCP's response to a domain he had ticked on the SNAP Tool: ‘[she asked] “what do you mean by this?”…She listened and we discussed it’ (P0111). Finally, although these HCPs acknowledged some tensions between delivering SNAP and wider organisational constraints (e.g. time, providing standard medical care and risk), they typically articulated how they tried to address these, rather than compromise the person-centred nature of SNAP. For example, one HCP described seeking to balance limited time to address support needs identified by one patient by offering ‘reassurance that we’ll talk about this [SNAP Tool question] today, but the next time we’ll be able to talk about other stuff [on the tool]’ (HCP023).

##### Deviating from SNAP as planned

In contrast, the remaining HCPs indicated reluctance to fully engage with SNAP, arguing that they did not need a means of delivering person-centred care as they ‘did this already’, ‘unpicking the issues as we’re going along’ (HCP014). Typically, their delivery of SNAP centred on the SNAP Tool itself, but with less fidelity to the intervention's five stages. As such, their patients were given the opportunity to complete the tool (although, as noted below, how it was sometimes introduced may have compromised this) and the patients’ responses were noted in the consultation, but few of their patients described SNAP delivery as intended. In particular, these patients rarely described HCP facilitation of a needs-led conversation or shared response, with some unable to recall a conversation or others simply commenting that the HCP had ‘a look at a few bits’ (P0714) or ‘did go through a few things’ (P0604). These patients rarely mentioned HCP use of open questions, empathy or consideration of patients’ views. Instead, their narratives suggested perception of greater use of closed or rhetorical questions, exemplified by one patient who recounted ‘[the HCP] opened it up looked at it and went “oh right …so there's not much you want to talk about”’ (P0114).

The unwillingness of these HCPs to deliver SNAP as intended was further illustrated by the accounts of ways in which their approach limited, rather than supported, patient engagement in SNAP. In particular, some patients described the SNAP Tool as being introduced to them as a ‘questionnaire’ or ‘survey’ leaving them unaware of the tool's role in enabling them to consider and have a conversation about their support needs. Others described not being sure what to do with their completed tool, or which HCP would discuss it with them. These HCPs often referred to their medical responsibilities’ and organisational constraints’ impacts on delivering SNAP but did not report using strategies to address these barriers, in contrast to HCPs who demonstrated fidelity to SNAP.

#### Theme 2: patient engagement with SNAP

Differences were also identified in how patients described engaging with SNAP. These differences related to patients’ perceptions of the SNAP Tool's purpose and relevance, and how they prepared for the SNAP conversation. This ‘engagement’ occurred along a continuum from active to passive. Three characterisable points on this continuum are discussed below: active, limited and passive.

##### Active engagement

On encountering the SNAP Tool, actively engaged patients recognised its potential in enabling them to identify and express their support needs to HCPs. For example, one commented ‘It [the SNAP Tool] asks all the right questions and some that are never asked…, you know, “relationships?”, “does a family member need support?”…these are probably things that never come up…they should, but they don’t’ (P111).

The SNAP Tool encouraged them to think more broadly about their support needs for example, one patient noted ‘[I] had never thought about it [the future], but seeing it on there you think “yeah, let's see what my future will hold”’ (P424). Others commented that the tool's questions legitimised raising issues with HCPs that they had not previously considered appropriate to discuss in a respiratory context, such as their anxiety or loneliness.

Consequently, these patients actively prepared for the SNAP conversation recounting, like this patient, ‘[taking] my time [to] fill that [the SNAP Tool] in because, you know, the questions on there I thought “well, you know, you need to think about them”’ (P323). Others described how completing the tool prompted thoughts around addressing their support needs that is, whether this was something they could do themselves or whether it would be useful to discuss with the HCP.

##### Limited engagement

Patients towards the middle of the continuum also understood and acknowledged SNAP's potential benefits. Reflecting on the SNAP Tool, one commented ‘it's an excellent booklet because people can see where they need help’ (P362); another noted ‘I think it's very useful [if you don’t have] the ability to know your way around [services] and be forceful or argumentative’ (P831). However, they differed from actively-engaged patients in how far they perceived the tool as (currently) applicable *to them*, as individuals. They typically commented that the questions ‘didn’t apply’ to them, or that addressing their support needs was ‘common sense’. Some also said they preferred the HCP to take the lead.

Despite these perceptions most limited-engagers completed the tool prior to the SNAP conversation and, like the actively engaged, noted how the tool guided them to identify their support needs from the comprehensive range of support domains it presented. However, unlike the actively engaged, they were less likely to describe using the tool as prompt to think more deeply about their support needs. This was exemplified by one patient who described how ‘it [the SNAP Tool] arrived in the post and I just quickly ticked it off’ (P214). Also, these patients rarely described the opportunity the tool gave them to raise and discuss their needs with an HCP. Instead, they often concluded that they did not have any support needs they currently wished to discuss, or that the tool provided an opportunity to raise an issue that they would have done anyway.

##### Passive engagement

For the remaining patients completing the SNAP Tool and preparing for the SNAP conversation was a process about which they had little understanding and, therefore, no investment. Instead, their response to the SNAP Tool appeared to be dictated by their perceptions of what their HCPs expected. For example, discussing these patients, one HCP noted: ‘they will happily partake in anything we give them. They are that sort of patient group…they will say “Oh the nurses have just asked me to fill it in so I will fill it in for them”’ (HCP014).

Typically, these patients did not understand that the tool was designed to enable them to identify their own support needs, or that it sat within a wider intervention. Instead, their accounts indicated that they thought they were completing a survey or filling in the tool to help the HCPs e.g. one patient described how the HCP: ‘presented the forms to me and asked if I could have a look at them and asked if I was happy to fill them in, and I have done that’ (P631).

The low engagement was manifest by the lack of time or attention these patients gave to tool completion, as one said: ‘I ticked it off and I thought, “Right I’ll tick it, tick it”…I didn’t think about nothing when I was doing it…just ticked it off…[…] just like a tax form’ (P254). Where these patients did think about the tool, they often misunderstood its purpose and completed it regarding supportive input already received rather where they needed more support. Furthermore, these patients had little anticipation that an HCP would look at, or respond to, any identified support domains, and consequently seemed to invest little time considering what they would like to discuss.

### Types of care

By exploring the interaction between the HCPs’ fidelity to the intervention (theme 1) and the three types of patient engagement (theme 2) we identified a continuum of care (from person-centred to HCP-led) enacted within SNAP.

#### Person-centred care

Where HCPs demonstrated intervention fidelity, their interactions within SNAP enabled a patient-led approach. Typically, this involved the patient identifying and expressing where they needed more support and a shared patient–HCP response to their prioritised support needs. For example, one patient, who identified needing help with getting out and about, was able to: (1) describe embarrassment when wearing an oxygen mask, (2) express how this stopped her accessing social activities, (3) identify that she needed and wanted support with this and (4) develop strategies with the HCP to address it. Developing a shared response (Stage 4) was harder with some patients, however patients found this process more interactive than usual care: many described discussing support they felt would be useful and being given opportunities to consider (and sometimes reject) HCP suggestions.

When these HCPs encountered limited-engagers, to encourage involvement they typically described taking time to ‘check there wasn’t anything else [the patient] wanted to talk about’ (HCP023), remaining faithful to the intervention. Some of these patients recounted how this led to them to engage fully with SNAP for example, one patient, who had not identified any support needs on the tool, described how they went through it with their HCP and ‘talked about each question […]…then I did think I would like to know a little bit more about “what to expect in the future”’ (P321). For others, reviewing the SNAP Tool with the HCP confirmed that they currently had no unmet support needs. However, passive patients could not recall taking part in SNAP, beyond completing the tool. Overall, however, these HCPs were delivering person-centred care.

Where SNAP was delivered (and engaged with) as intended, benefits were identified by both patients and HCPs such as opening new areas of discussion, with one HCP reflecting: ‘people have come in with questions that it wouldn’t have occurred to me to ask’ (HCP011). SNAP also enabled more in-depth discussion of known concerns for example, a patient with known depression, commented how: ‘The SNAP Tool prompted me to ask some more questions [about my depression]. That was something that I hadn’t understood in the past – that I should have asked more relevant questions’ (P111). HCPs were surprised when patients highlighted needing more support with understanding their illness or dealing with their feelings, which the HCPs felt they had already addressed. Both HCPs and patients noted that SNAP was ‘definitely really helpful with end-of-life conversations’ (HCP013).

As a result of this person-centred approach, most of these patients described shared response to their need. For some patients, SNAP facilitated the opportunity to talk to the HCP, find out more about their condition or discuss its management. For others, SNAP enabled access to supportive input beyond traditional medical responses for example, referral to befriender schemes, peer support or community groups.

#### HCP-led care

Where HCPs deviated from delivering SNAP as intended, HCP–patient interactions within SNAP were more aligned with a traditional bio-medical response.

With those patients who had actively engaged with the SNAP Tool, and identified that they needed more support, these HCPs then typically undertook an HCP-led needs assessment and responded with HCP-determined supportive input. For example, one patient described using the SNAP Tool to express that he needed more support in ‘knowing what to expect in the future’ (P0204), whereas his HCP concluded his ‘primary need was actually to understand his condition’ and so ‘went through education on COPD [with him]’ (HCP024).

When these HCPs encountered limited or passively engaged patients, their narratives suggested these patients’ responses were taken at face value; patients were not encouraged to become engaged with SNAP. In particular, passive patients of these HCPs reported either not having a conversation about the SNAP Tool or, like this patient, that they couldn’t ‘remember if [the HCP] did go through that [the SNAP Tool] with me or not’ (P534). Some patients of these HCPs used the SNAP Tool to make simple requests traditionally associated with HCPs’ usual role and the medical context (e.g. requesting a letter of support for a benefit claim or checking inhaler technique).

Apart from patients who had no engagement with SNAP, most patients receiving HCP-led care were still pleased to have had an opportunity to highlight their support needs through the SNAP Tool and access supportive input. For example, one reflected positively on how the HCP had said that the team ‘would back me up [with her housing claim]’ (P714). In contrast, HCPs delivering HCP-led care felt that SNAP (as they delivered it) did not add value to their practice concluding ‘we’ve been covering those issues anyway’ (HCP014) and ‘at any stage in the clinic that conversation would still come up’ (HCP024). Some of these HCPs also perceived that the patients had only identified a support need because they felt ‘they’d better tick something’ (HCP034). Another commented that, as the patients had attended for a specific medical purpose, they ‘don’t want to be there even longer discussing…things that have already been discussed’ (HCP014).

The care enacted within SNAP was not, however, dichotomous: person-centred or HCP-led care occurred along a continuum reflecting the fact that HCPs’ fidelity to the intervention and the three types of patient engagement were not themselves discrete categories.

## Discussion

This pilot study explored the patient and HCP experiences of a new intervention operationalising delivery of holistic person-centred care (SNAP) to establish whether, and how, it supported patient identification, expression and discussion of unmet support needs. There were differences in how HCPs delivered SNAP and how patients engaged with it; analysing the interaction of these identified a continuum of care (from person-centred to HCP-led) which impacted patient identification and expression of need and resulting responses.

When delivered as intended, SNAP supported patient-led identification and expression of their support needs for patients who actively engaged with it, with similar findings for patients who were limited-engagers but encouraged by their HCP to revisit the SNAP tool together. Patient-completion of the tool legitimised raising their support needs with HCPs, enabled patients to articulate directly to HCPs where they needed more help, and made the process visible. The SNAP conversation was then centred on patient-identified and prioritised areas of support need: it enabled exploration of the specific support needs and a co-developed response. As a result, these patients received a range of supportive inputs including the opportunity to talk, reassurance, future care planning and access to medical and non-medical services. Some patients identified no current need for support, but the opportunity to review this with an HCP ensured this remained a person-centred consideration of their current circumstances rather than reflecting a lack of understanding of SNAP's purpose. These HCPs were delivering (and these patients receiving) person-centred care.

Where SNAP was not delivered as intended, patients were far less likely to identify and express their support needs: fewer patients fully engaged with the SNAP Tool or understood SNAP's potential utility. Critically, both passive and limited-engagers indicating no support need appeared less likely to be encouraged to re-consider the SNAP Tool and truly consider their support needs. This may represent missed opportunities by these HCPs to identify and address unmet support needs as there was uncertainty about whether this was a patient-led choice not to engage. Where patients did express their support needs to HCPs who deviated from SNAP the follow-on discussion was more akin to usual care in terms of content and outcome for example, traditional bio-medical, rather than holistic, responses. These HCPs were delivering (and these patients receiving) HCP-led care.

These results suggest firstly that, if delivered as intended, SNAP enables patients to overcome well-established personal, institutional and organisational barriers to identifying and expressing need.^22–26^ Kendal et al.^22^, Beernaert et al.^23^ and Coventry et al.^24^ note patient reluctance to raising their concerns due to feeling that it is inappropriate within a medical consultation, desire for independence or limited awareness of needs. Even if raised, Chew-Graham et al.^25^ and Chatwin et al.^26^ found that patient concerns and expressions of need can be curtailed by the focus HCPs can place on institutional or medical concerns. Our findings suggest that SNAP addresses these barriers by making visible, and legitimising, the support needs patients can discuss with their HCP and by providing a mechanism for HCPs to deliver person-centred care.

Secondly, the findings demonstrate the capability of the designed-for-purpose SNAP Tool to enable *direct expression of unmet support needs*. This overcomes the limitations identified in the literature by McElduff^9^ and Osse^10^ regarding symptom/burden/problem-based instruments which only *indirectly indicate* a need for support.

Thirdly, however, our findings also underline that understanding SNAP as more than just the SNAP Tool is essential. SNAP is a five stage, two-component intervention: the SNAP tool (Stages 1 and 2), plus the needs-led conversation that follows tool completion (Stages 3+). Where the stages of SNAP were delivered as intended, person-centred care was achieved in the identification, discussion and response to support needs. In contrast, within the HCP-led approach, patient involvement centred on completion of the SNAP Tool resulting in a process vulnerable to traditional HCP interpretations and assumptions. To achieve person-centred care through SNAP, HCP understanding of the intervention as a process (beyond SNAP Tool completion) is essential. As an intervention, SNAP provides an enhanced alternative to tools such as the Supportive Care Needs Survey^9^ which, similar to the SNAP Tool, enables patients to identify their support needs, but, unlike SNAP (the intervention), does not support HCPs to then involve patients in a needs-led process beyond the survey responses. It is also noteworthy, however, that some patients who received SNAP as HCP-led care also gave positive feedback, reflecting findings^11^ that patients can respond positively to the use of tools within consultations even in the absence of person-centred care.

Finally, the findings confirm the pivotal role intervention fidelity plays in delivering person-centred care via SNAP. Notably, despite being highly experienced with long-established relationships with many of her patients, one nurse (who demonstrated high intervention fidelity) still felt that SNAP supported delivery of a more person-centred approach than her usual care. Study learnings will therefore inform the enhancement of SNAP training including greater understanding of person-centred care, of SNAP as a process, patient–HCP interactions that can occur when using SNAP and what HCPs need to do to deliver SNAP effectively.

### Strengths and limitations

A key study strength was its access to accounts from patients and HCPs across settings, enabling the exploration of a range of perspectives and interactions. In addition, HCPs delivered SNAP to 56 patients and were therefore able to reflect on their experiences with a relatively large sample for a pilot study.

A potential limitation was that all clinical settings were in the East of England. Also, this analysis included only one site per setting (primary, community and secondary care) limiting our ability to explore the influence of settings on different approaches to delivering SNAP; furthermore, only one HCP delivered SNAP in primary care. Patient engagement with SNAP may also have been limited by the study itself: HCPs reported that some patients declined to complete the SNAP Tool (and therefore receive SNAP) due to reluctance to participate in research. As a result, our findings may not fully reflect intervention engagement in a non-experimental setting. Finally, SNAP was developed by three of the authors who also conducted this pilot study.

## Conclusion

The SNAP^12,13^ operationalises delivery of holistic person-centred care, providing an alternative to HCP-led approaches to identifying and addressing patient support needs. When delivered as intended, it enables identification, expression and discussion of support needs by legitimising support need expression and making care visible. SNAP addresses the rhetoric within policy, good practice guidance and the person-centred care literature espousing the need to involve patients in identifying their needs, goals and preferences by providing HCPs with a mechanism for how a truly holistic person-centred approach can be achieved in everyday practice.
